# Drug-Related Problems in Patients with Benign Prostatic Hyperplasia: A Cross Sectional Retrospective Study

**DOI:** 10.1371/journal.pone.0086215

**Published:** 2014-01-27

**Authors:** Hasniza Zaman Huri, Chong Hui Xin, Che Zuraini Sulaiman

**Affiliations:** 1 Department of Pharmacy, Faculty of Medicine, University of Malaya, Kuala Lumpur, Malaysia; 2 Clinical Investigation Centre, University Malaya Medical Centre, Kuala Lumpur, Malaysia; 3 Department of Pharmacy, University Malaya Medical Centre, Kuala Lumpur Malaysia; The Chinese University of Hong Kong, Hong Kong

## Abstract

Benign Prostatic Hyperplasia (BPH) patients are at risk of acquiring drug-related problems (DRPs), as it is present in the majority of aging men. To date, DRPs among BPH patients have not been well studied. We conducted this retrospective study in a tertiary hospital in Malaysia from January 2009 to June 2012 with the aim of identifying the factors associated with DRPs among BPH patients. The Pharmaceutical Care Network Europe Classification Version (PCNE) 5.01 was used as a tool to classify DRPs. We enrolled 203 patients from 259 hospital admissions. A total of 390 DRPs were found and there was an average of 1.5±1.3 problems per hospitalization. 76.1% of hospital admissions included at least one DRP. The most common DRP categories encountered were drug choice problems (45.9%), drug interactions (24.9%), and dosing problems (13.3%). Factors such as advanced age (p = 0.005), a hospital stay of more than 6 days (p = 0.001), polydrug treatments (p<0.001), multiple comorbidities (p<0.001), and comorbid cardiovascular disease (p = 0.011), diabetes mellitus(p = 0.001), hypertension (p<0.001) and renal impairment (p = 0.011) were significantly associated with the occurrence of DRPs. These data indicated that the prevalence of DRPs is high among BPH patients. The identification of different subtypes of DRPs and the factors associated with DRPs may facilitate risk reduction for BPH patients.

## Introduction

Benign Prostatic Hyperplasia (BPH) is a common disorder, which refers to the proliferation of stromal and epithelial cells within the prostate [1]. BPH is usually associated with a series of lower urinary tract symptoms (LUTS), including nocturia, increased urinary hesitancy, frequency and urgency, as well as a weak dribbling stream of urine and increased post-voiding residual volumes. The prevalence of BPH is progressive and increases linearly with age [1].The American Urological Association has demonstrated that symptomatic BPH affects 50% of men at the sixth decade of life and the prevalence increases to up to 90% of men aged above 85 years [2]. Likewise, the Ministry of Health of Malaysia (MOH) has reported that the prevalence of BPH in men aged above 60 years is about 50% and rises to up to 82% of men aged 71 to 80 years [3].

BPH is not a life-threatening disease but it can have detrimental impacts on quality of life, and untreated BPH can potentially result in complications such as acute urinary retention (AUR) and the need for prostate-related surgical interventions [4]. Transurethral Resection of the Prostate (TURP) was the most common treatment for BPH during the last decade. However, α_1_-adrenergic blockers and 5α-reductase inhibitors have been accepted as standard medical therapies for BPH since the 1990s, as they are approved to improve urinary functions in men with BPH [5].

BPH is present in majority of the aging men, who have an age of around 60years and above[4].This population is more vulnerable to drug-related problems (DRPs) [6].A study conducted by Boyle and Napalkov revealed that there is a higher prevalence of hypertension in patients with BPH, because of increased prostate gland volumes that will subsequently increase diastolic blood pressure [7].Also, because of the high prevalence of multiple comorbidities in older populations, patients with BPH are more likely to be prescribed multiple medications, leading to increased risks of drug-drug interactions [8]. Apart from that, age-related alterations in pharmacodynamics and pharmacokinetics of drugs, which might potentiate or reduce their efficacies, can lead to the occurrence of DRPs [9].

DRP is a term describing, “an event or circumstance involving drug therapy that actually or potentially interferes with desired health outcomes” [10]. Undetected DRPs may result in drug-related morbidity and if unattended or untreated, it may lead to drug-related mortality. In addition to that, DRPs can have substantial impacts on the economy, as the cost of care attributable to drug-related morbidities is high [11]. Nonetheless, DRPs are usually preventable. Health care providers, especially pharmacists, are in a position in health care settings to recognize and prevent DRPs, as well as to reduce drug-related morbidity and mortality [12].

Up to now, the DRPs associated with BPH have not been well studied. Similarly, because of the lack of local study of BPH in Malaysia, DRPs in patients with BPH in this country have not yet been identified. Therefore, we conducted this study to evaluate whether they are subtypes of DPRs and the factors associated with DRPs in patients with BPH.

### Objectives

1. To investigate the types and causes of drug-related problems in patients with benign prostatic hyperplasia.
2. To identify factors associated with the drug-related problems in patients with benign prostatic hyperplasia.

## Methods

### Study Design and Setting

This was a retrospective study conducted in a premier teaching hospital in Malaysia with 1000 beds, which was the University of Malaya Medical Centre (UMMC), Malaysia. This study was conducted in accordance to Declaration of Helsinki and was approved by the medical ethics committee (MEC) of UMMC (reference number 956.34). The MEC of UMMC waived the need for written informed consent from the participants.

### Study Population and Sampling Frame

The minimum sample size was calculated using Epi Info Program Version 7.0 (CDC, Clifton Rd. Atlanta, USA).The level of significance, α, was set as 0.05 and the desired power of the study, 1-β, was 80%. Assuming that the expected proportion of patients with BPH was 50% and the confidence limit was 5%, the minimum sample size calculated was 164 patients [2], [3]. The study population consisted of all BPH patients who fulfilled the requirements of the International Classification of Diseases Tenth Revision (ICD-10) code N40 and who were admitted to UMMC from the 1^st^of January 2009 to the 30^th^of June 2012.

Inclusion criteria:

- Adult patients who were aged above 18 years.
- Patients who were currently diagnosed with BPH.
- Patients had been prescribed at least one medication indicated for BPH.

Exclusion criteria:

- Patients with missing data.

### Data Collection

Data was collected by the authors, who are all pharmacists.

- Patients' demographic information such as age, ethnic, weight, height and duration since the patients were diagnosed with BPH.
- Co-morbid medical conditions [13].
- BPH complications such as acute urinary retention, bladder stones, gross hematuria, and urinary tract infection [14], [15].
- Laboratory results and other monitoring parameters as stated in medical reports.
- Drug therapy including treatment of BPH and other concurrent medications.
- Assessment of DRPs based on the PCNE Classification V5.01 [10].

Table 1 shows the definition used in the study [16]–[28].

**Table 1:** Definitions used in the study.

Terms	Definition
Acute urinary retention	A clinical condition that is characterized by sudden onset of pain and inability to urinate [16].
Bladder stone	A condition where there are small masses of minerals formed in the bladder, as a result of urinary stasis [17].
Benign Prostatic Hyperplasia	A progressive disorder, which appears to be a common problem among the majority of men aged 60 years and above [14]. Patients with BPH are normally presented with a variety of lower urinary tract symptoms, LUTS, which can be categorized as either obstructive or irritative symptoms [18]. Obstructive symptoms are associated with urinary hesitancy, weak urine stream, urine dribbles out of penis, incomplete bladder emptying as well as increased post-void residual volume. In contrast, patients with irritative symptoms often experience an increase in frequency, urgency and nocturia [19].
Cardiovascular disease	Disorders of the heart and blood vessels, involving coronary heart disease, stroke, hypertension, peripheral artery disease, rheumatic heart disease, congenital heart disease and heart failure, or as stated in the medical records [20].
Cerebrovascular accident	A condition in which a clot interrupts the blood flow to the brain or blood vessels in the brain burst, or as stated in the medical records [21].
Diabetes mellitus	A chronic disease that can be caused by inadequate production of pancreatic insulin (Type I) or ineffective use of insulin (Type II) [22].
Hematuria	It includes macroscopic hematuria, which is the presence of blood in the urine that can be seen by naked eyes, and microscopic hematuria, where the presence of blood is only visible under microscope [23].
Hepatic impairment	Refers to alcoholic or non-alcoholic liver cirrhosis, drug-induced hepatotoxicity, chronic hepatitis, liver cancer, increase of the serum liver enzymes for more than three times of the normal upper limits, or as stated in the medical records [24].
Hypertension	A condition in which the pressure of the blood vessels is persistently high, where systolic blood pressure ≥140 mmHg and diastolic blood pressure ≥90 mmHg, or as stated in the medical records [25].
Polypharmacy	The use of at least five medications [26].
Renal impairment	A condition that includes acute kidney injury, which refers to the sudden and rapid reduction of renal functions, over hours to days, as well as chronic kidney injury, which refers to the deterioration of kidney functions for more than three months, or as stated in the medical records [27].
Urinary tract infection	An infection that can affect either lower or upper urinary tract. Lower UTI involves the bladder while upper UTI involves the kidneys [28].

### Classifications and Assessment of DRPs

Pharmaceutical Care Network Europe (PCNE) classification of DRPs version 5.01 [10] was used to categorize DRPs. It is an established system that has been revised several times and its validity and reproducibility have been tested [29], [30]. It had been used by many recent studies [31], [32], [33]. In this study, the six domains of problems included in the PCNE classification were used. The DRPs and their possible causes were identified from the patients' medical records, with reference to the standard guidelines and established literature [1]–[3], [5], [34], [35]. All authors wereinvolved in the identification and classification of DRPs.

### Updated Beers Criteria

The American Geriatrics Society (AGS) 2012 Beers Criteria serves as a tool to assess the appropriate use of medications among elderly patients. It consists of a drug list involving the drugs that should be avoided in older adults, drugs to be avoided in older patients with certain diseases, and drugs to be used cautiously in geriatric patients. In this study, the Beers Criteria were used to assess whether drugs were inappropriately prescribed to BPH patients for the items included in P2.1, P2.2, and P2.4 in the drug choice problem domain of PCNE V5.01. Only listed drugs with ‘strong strength of recommendation’ were recognized as DRPs in this study.

### Statistical Techniques

All of the collected data were pooled and analyzed using IBM SPSS Statistics Version 20.0 (Armonk, New York, USA). Descriptive statistics were used to summarize patients' demographic and clinical characteristics. Frequency tables were used to tabulate different types of DRPs and the causes of these DRPs. Continuous data were expressed as the mean±standard deviation whereas categorical data were expressed at a percentage. The relationships between categorical variables were examined using Pearson Chi-square tests with Continuity Correction and Fisher's Exact tests when necessary. Significance α was accepted at p<0.05.

## Results

### Demographic Characteristics

**Figure 1:**
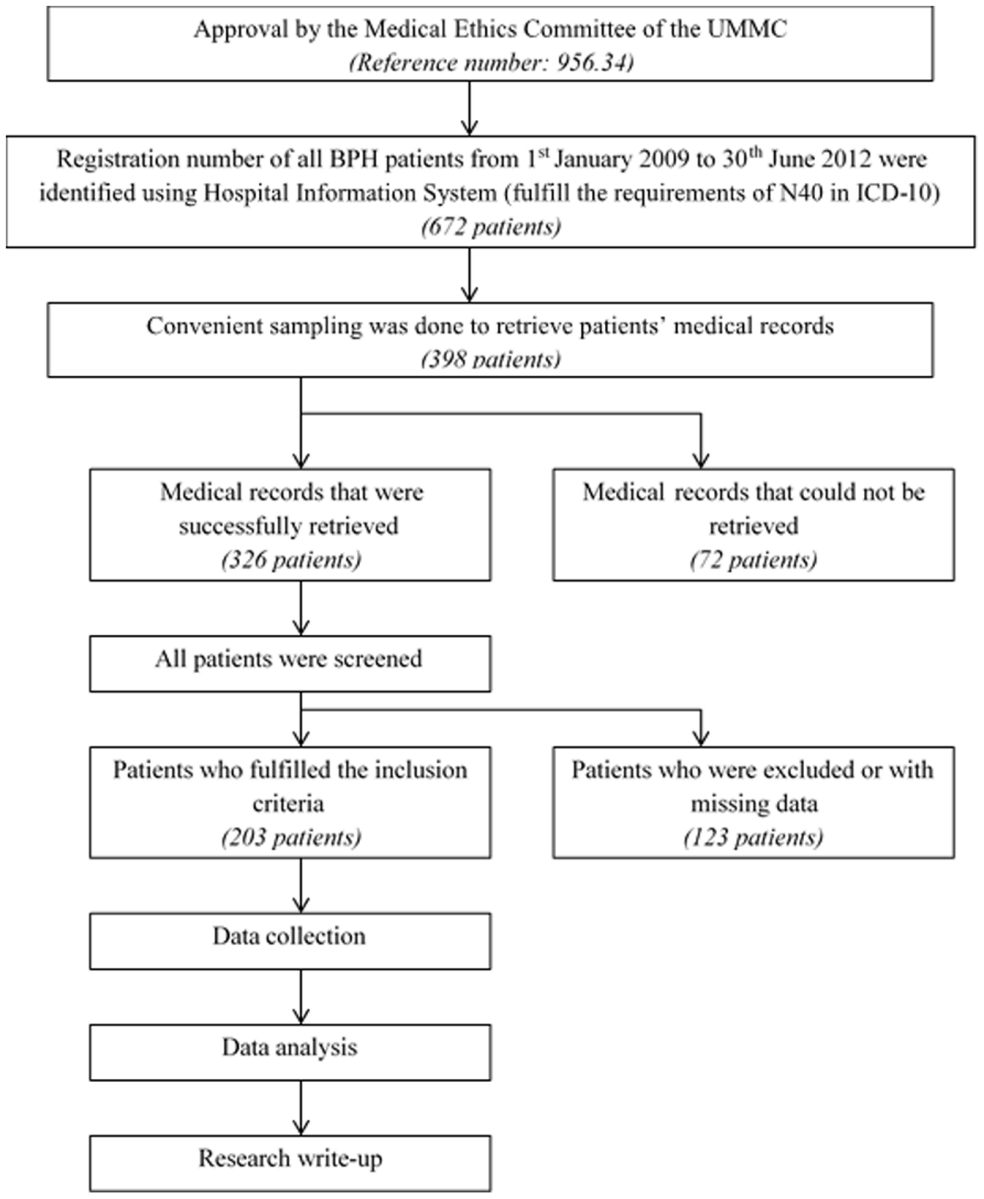
Overview of methodology.

Overall, 203 patients were enrolled in this study and there were a total of 259 admissions to the hospital. Of these 203 patients, 45 (22.2%) experienced more than one hospitalization. Figure 1 shows the demographic characteristics of the patient population. The majority of them were at the age of 60 to 79 years old. The minimum and maximum age of the patients were 48 and 96 years old, respectively, whereas the median age of the study population was 71 years old. Elderly patients comprised more than three quarters (77.8%) of the study population and the number of elderly patients appeared to be greater than that of non-elderly patients, in both groups of patients, with DRPs and without DRPs (refer to Table 2).

**Table 2:** Demographic characteristics of patients with BPH (N = 203).

Number (Percentage, %)	TotalN = 203 (100.0%)	With DRPN = 134 (66.0%)	No DRPN = 69 (34.0%)
**Demographic characteristics**			
Age, years	70.7±8.4	71.9±8.3	68.5±8.1
**Age group**			
Non-elderly	45 (22.2)	19 (14.2)	26 (37.7)
Elderly	158 (77.8)	115 (85.8)	43 (62.3)
**Ethnicity**			
Malay	60 (29.6)	37 (27.6)	23 (33.3)
Chinese	104 (51.2)	70 (52.2)	34 (49.3)
Indian	35 (17.2)	24 (17.9)	11 (15.9)
Others	4 (2.0)	3 (2.2)	1 (1.4)
**Body Mass Index (BMI)**			
Underweight (<18.5)	33 (16.3)	29 (21.6)	4 (5.8)
Normal range (18.5–22.9)	45 (22.2)	32 (23.9)	13 (18.8)
Pre-obese (23.0–27.4)	16 (7.9)	8 (6.0)	8 (11.6)
Obese (27.5–34.9)	13 (6.4)	9 (6.7)	4 (5.8)
Unknown	96 (47.3)	56 (41.8)	40 (58.0)

Note: BMI = Weight (kg)/[Height×Height (m^2^)].

### Clinical Characteristics

Of the 259 admissions, the mean duration of hospitalization was 7.1±10.2 days. Approximately 38% of the patients stayed for more than six days in the ward, with the longest duration being106 days. On the other hand, more than 60% of the patients stayed for less than six days in the ward and the minimum period of hospitalization was one day. About half of the patients were diagnosed with BPH for less than five years whereas only 16.7% of them had BPH for more than five years (refer to Table 3).

**Table 3:** Clinical Characteristics of Patients with BPH (N = 203).

Number (Percentage, %)	TotalN = 203 (100.0%)	With DRPN = 134 (66.0%)	No DRPN = 69 (34.0%)
**Duration of hospitalization**			
Less than six days	126 (62.1)	76 (56.7)	50 (72.5)
More than six days	77 (37.9)	58 (43.3)	19 (27.5)
**Duration of BPH**			
Less than five years	99 (48.8)	61 (45.5)	38 (55.1)
More than five years	34 (16.7)	22 (16.4)	12 (17.4)
Unknown	70 (34.5)	51 (38.1)	19 (27.5)
**BPH complications**			
Acute urinary retention	35 (17.2)	26 (19.4)	9 (13.0)
Renal stones	23 (11.3)	13 (9.7)	10 (14.5)
Hematuria	42 (20.7)	30 (22.4)	12 (17.4)
Urinary tract infection	35 (17.2)	30 (22.4)	5 (7.2)
**Comorbidities**			
Cardiovascular disease	58 (28.6)	48 (35.8)	10 (14.5)
Cerebrovascular accident	24 (11.8)	23 (17.2)	1 (1.4)
Diabetes mellitus	81 (39.9)	61 (45.5)	20 (29.0)
Dyslipidemia	47 (23.2)	32 (23.9)	15 (21.7)
Hypertension	130 (64.0)	94 (70.1)	36 (52.2)
Erectile dysfunction	2 (1.0)	1 (0.7)	1 (1.4)
Gouty arthritis	9 (4.4)	6 (4.5)	3 (4.3)
Bronchial asthma	21 (10.3)	19 (14.2)	2 (2.9)
Renal impairment	33 (16.3)	29 (21.6)	4 (5.8)
Hepatic impairment	7 (3.4)	5 (3.7)	2 (2.9)

Note: A patient may have more than one BPH complication or comorbidity.

The most common BPH complication associated with these patients was hematuria, which occurred in20.7% of the study population. This was followed by acute urinary retention and urinary tract infection, which occurred in 17.2% of the study population, respectively. Only 11.3% of the patients had experienced renal stones. On average, the patients had 2.3±1.6 comorbidities. Approximately two thirds (64%) of the BPH patients had comorbid hypertension, 39.9% had comorbid diabetes mellitus, and 28.6% of them had comorbid cardiovascular disease. These chronic diseases made up the three most common comorbidities in BPH patients. The mean number of medications prescribed in each hospitalization was 8.1±4.8 drugs. Polydrug therapy was seen in 74.1% of all the admissions and 83.9% of polydrug-therapy-treated patients were elderly.

### Drug Treatments Used in BPH Patients

#### BPH medications

**Figure 2:**
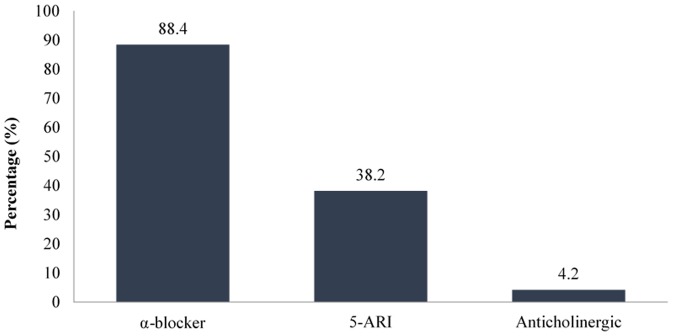
Classes of BPH medications used during hospitalization (N = 259) Note: A patient may receive more than one BPH medication.

Monotherapy BPH medications were most commonly prescribed medications in this study, representing approximately 70% of the 259 admissions. Dual therapy, which consists of either the combination of an α-blocker and a 5α-reductase inhibitor (5-ARI) or an α-blocker and an anticholinergic (tolterodine) or a 5-ARI and an anticholinergic was used in 29.3% of the total admissions. In contrast, a triple regimen of an α-blocker, a 5-ARI, and an anticholinergic was only prescribed to 0.8% of the patients. Figure 2 shows the use of BPH medications during the hospitalizations.

Four types of α-blockers were prescribed in the study population. Doxazosin (57.2%) was more commonly used than alfuzosin (38.4%). Terazosin and tamsulosin were the least commonly prescribed agents, as they were only prescribed in 2.6% and 1.7% of the study population, respectively. In contrast, of the 99 cases of hospitalization that involved prescription of 5-ARIs, finasteride (59.6%) appeared to be a more common choice compared to dutasteride (39.4%), whereas the combination of dutasteride and finasteride was used only in one (1.0%) hospital admission.

### Other Concurrent Medications

**Figure 3:**
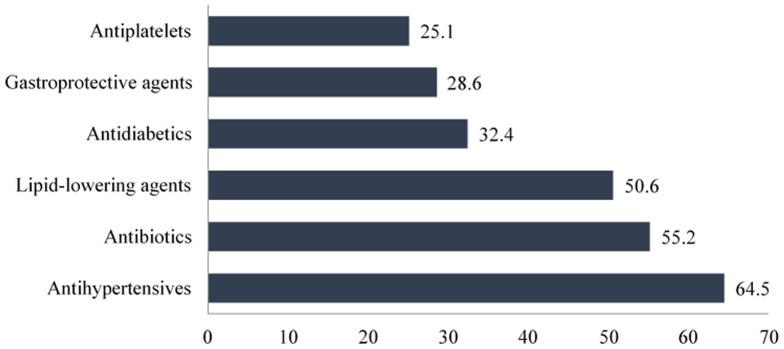
Classes of concurrent medications that were most frequently implicated during hospitalization (N = 259).

Figure 3 reveals the six most frequently implicated drug classes being used other than the BPH medications. In total, antihypertensive agents, antibiotics, and lipid-lowering agents were implicated in more than 50% of the hospitalizations. A calcium channel blocker (CCB) was the most commonly (32.5%) given antihypertensive agent in the study population. This was followed by angiotensin-converting enzyme (ACE) inhibitors (20.4%), diuretics (16.8%), beta-blockers (16.4%), and angiotensin receptor blockers (ARBs) (11.4%). Alpha-blockers such as prazosin was the least commonly used (2.5%) antihypertensive agent.

Cephalosporin was to be the most widely used antibiotic, making up 37.9% of all the cases of hospitalization. This was followed by the other three classes of antibiotics, namely fluoroquinolone (27.8%), penicillin (20.1%), and carbapenem (5.9%). Antibiotics that did not fall into the four classes above were classified as “others”, including macrolides, glycopeptide, aminoglycoside, nitrofurantoin, and metronidazole. On the other hand, two classes of lipid-lowering agents were prescribed to the study population, which were HMG-CoA reductase inhibitors (statins) and fibrates. Statins were prescribed in more than 99% of the patients, whereas fibrates was only used in one case of hospital admission.

### DRPs in BPH Patients

**Figure 4:**
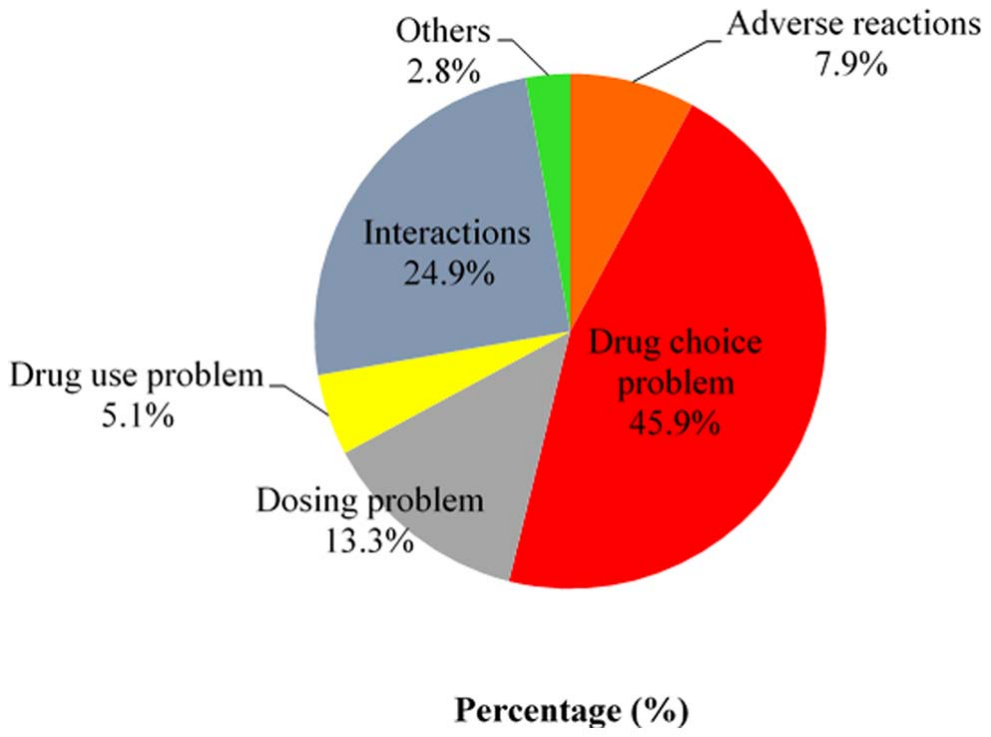
Drug-related problems in patients with BPH (n = 390).

A total number of 390 DRPs were identified in this study. There was an average of 1.5±1.3 problems and 1.1±1.0 causes of DRPs per hospital admission. 197 (76.1%) cases of hospitalization were detected, with at least one clinically relevant DRP, and a maximum of six DRPs in any single patient. The top three categories of DRPs encountered were drug choice problems (45.9%), drug interactions (24.9%), and dosing problems (13.3%) as shown in Figure 4. This was followed by adverse reactions (7.9%) and drug use problem (5.1%), whereas the “others” was the least identified problem, which composed only 2.8% of all the DRPs.

### Adverse Reactions

A total of 31 adverse reactions involving non-allergic (7.1%), allergic (0.3%), and toxic effect (0.3%) reactions to the drugs were identified. Of these, α-blockers such as alfuzosin, doxazosin and tamsulosin that were prescribed to treat BPH were implicated in three cases of postural hypotension. Antidiabetic agents such as insulin and sulphonylureas were associated with six cases of hypoglycemia in this study. In contrast, antihypertensive agents such as perindopril, hydrochlorothiazide, and spironolactone were associated with three cases of adverse reactions, which were 1 case of persistent dry cough, 1 case of hyponatremia, and 1 case of gynaecomastia. It has been reported that antiplatelets such as aspirin, clopidogrel, and ticlopidine cause bleeding disorders, including both hematoma and hematuria. In our study, aspirin was associated with one case of anaphylaxis. Other drug classes we examined such as antibiotics, anticoagulants, analgesics, and psychotropic agents were also reported to cause adverse reactions in this study.

### Drug Choice Problems

As shown in Table 4, drug choice problems that were most frequently recorded were inappropriate drug choice, untreated indications, and no clear indication for drug use, which contributed to more than 35% of all DRPs.

**Table 4:** Drug choice problems in patients with BPH (n = 390).

Code	Problems	Number of problems (Percentage, %)
**P2**	**Drug choice problems**	**179 (45.9)**
P2.1	Inappropriate drug (not most appropriate for indication)	67 (17.2)
P2.2	Inappropriate drug form (not most appropriate for indication)	3 (0.8)
P2.3	Inappropriate duplication of therapeutic group or active ingredient	8 (2.1)
P2.4	Contraindication for drug	29 (7.4)
P2.5	No clear indication for drug use	34 (8.7)
P2.6	No drug prescribed but clear indication	38 (9.7)

In this study, prescriptions of several drugs in elderly patients were considered as inappropriate medication use according to the Updated Beers Criteria. These drugs accounted for most of the inappropriate drug choices identified, including metoclopramide (23 cases), ticlopidine (15 cases), first generation antihistamines (11 cases) such as chlorpheniramine, diphenhydramine and triprolidine, sliding scale insulin (8 cases), and prazosin as a first line antihypertensive (7 cases).

There were 38 cases where no drugs were given despite clear indications. For instance, symptomatic anemia was left untreated in patients, especially those with renal impairments (13 cases), prophylaxis was not given for patients with high risk of gastrointestinal bleeding (8 cases), and treatment was not given to correct imbalanced electrolytes (6 cases).Besides that, there were cases where persistently high blood pressure was left unattended, there was untreated pedal edema, and aspirin was not given as a secondary prophylaxis in patients with a high risk of recurrent stroke.

On the other hand, 34 cases of drug choice problems involving the use of drugs without clear indications were recorded. These included the use of gastro-protective agents such as omeprazole and ranitidine for stress ulcer prophylaxis when there was no clear indication (11 cases) and the prescription of metoclopramide to prevent emesis when there was no active complaint from patients (9 cases).

### Dosing Problems

Table 5 shows the dosing problems in patient with BPH. Of these, BPH medications such as alfuzosin, doxazosin and dutasteride were related to three cases of dosing problems. Drugs that were most frequently prescribed at excessive dose were ranitidine (13 cases), antibiotics (11 cases), metoclopramide (6 cases), digoxin (3 cases) and others. Prescription of drugs at excessive dose was most commonly detected in patients with impaired renal functions, which comprised more than 90% of the cases.

**Table 5:** Dosing problems in patients with BPH (n = 390).

Code	Problems	Number of problems (Percentage, %)
**P3**	**Dosing problems**	**52 (13.3)**
P3.1	Drug dose too low or dosage regime not frequent	14 (3.6)
P3.2	Drug dose too high or dosage regime too frequent	33 (8.5)
P3.3	Duration of treatment too short	4 (1.0)
P3.4	Duration of treatment too long	1 (0.3)

### Drug Use Problems

There were a total of 20 (5.1%) drug use problems (drugs not taken/administered at all) that were identified in this study. BPH medication was implicated in nine cases of drug use problems in this study. It was reported that patients were not adhered to doxazosin and dutasteride in eight cases and one case of hospital admissions respectively. Also, antidiabetic agents such as gliclazide and insulin (3 cases), antihypertensive agents such as prazosin and diuretics (2 cases), antiplatelets including aspirin and ticlopidine (2 cases) as well as the other medications such as statins, allopurinol, and antibiotics were found to be associated with non-compliance of medications.

### Drug Interactions

**Figure 5:**
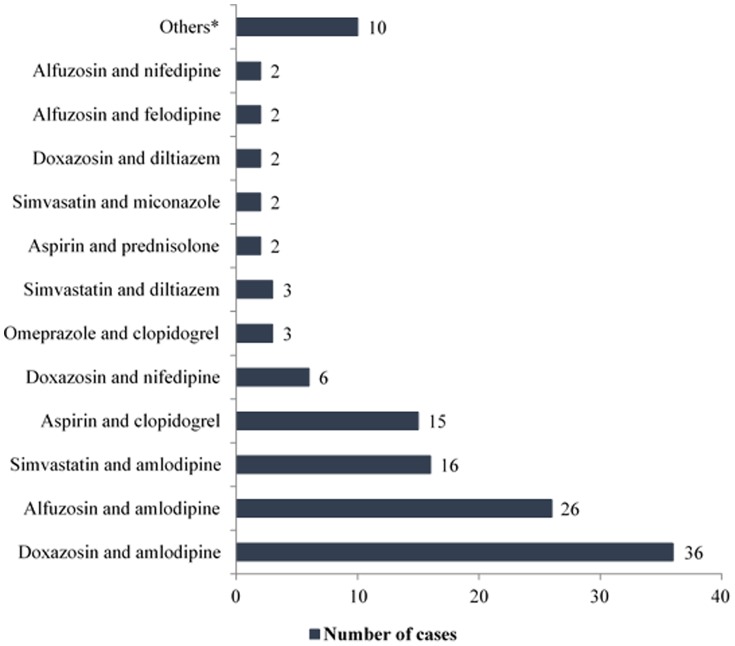
Drug pairs that were most commonly associated with drug interactions in patients with BPH (n = 35).

Among the 390 DRPs identified, approximately 25% of that was caused by potential drug-drug interactions.α-blockers that were used for the treatment of BPH were involved in more than half of all the drug interactions. The drug pairs that were most commonly to be associated with drug interactions were doxazosin and amlodipine (36 cases), alfuzosin and amlodipine (26 cases), simvastatin and amlodipine (16 cases), where simvastatin was prescribed at a dose greater than 20 mg/day, aspirin and clopidogrel (15 cases), as well as the other interactions, as shown in Figure 5. Only drug interactions that are classified as “moderate” or “severe” were identified as DRP in this study.

### Causes of DRPs in BPH Patients

Table 6 shows the causes of the 390 DRPs. A total number of 295 causes were identified. The number of causes reported differed from that of the number of problems, as some DRPs were associated with at least one cause whereas the others did not have any cause. Among the six domains of causes examined, inappropriate drug selection or dose selection and drug use process were the most common causes of DRPs in this study, which comprised 84.1% and 9.5% of all of the causes, respectively. Inappropriate drug selection or dose selection was most frequently associated with DRPs such as adverse reactions, drug choice problems, dosing problems, and drug interactions. In contrast, drug use problems were found to result from causes such as “drug use process” and “patient or psychological” parameters. However, some dosing problems were reported to be caused by logistic problems.

**Table 6:** Causes of DRPs in patients with BPH (n = 295).

Code	Causes	Number of causes (Percentage, %)
C1	**Drug/Dose selection**	**248 (84.1)**
C1.1	Inappropriate drug selection	128 (43.4)
C1.2	Inappropriate dosage selection	52 (17.6)
C1.4	Pharmacokinetic problems, including ageing/deterioration in organ function and interactions	1 (0.3)
C1.5	Synergistic/preventive drug required and not given	35 (11.9)
C1.7	New symptom or indication revealed/presented	4 (1.4)
C1.8	Manifest side effect, no other cause	28 (9.5)
C2	**Drug use process**	**28 (9.5)**
C2.1	Inappropriate timing of administration and/or dosing intervals	6 (2.0)
C2.2	Drug underused/under-administered	17 (5.8)
C2.3	Drug overused/over-administered	3 (1.0)
C2.5	Drug abused (unregulated overuse)	1 (0.3)
C2.6	Patient unable to use drug/form as directed	1 (0.3)
C3	**Information**	**1 (0.3)**
C3.1	Instruction for use/taking not known	1 (0.3)
C4	**Patient/Psychological**	**9 (3.1)**
C4.1	Patient forgets to use/take drug	2 (0.7)
C4.3	Patient suspects side effect	1 (0.3)
C4.5	Patient unwilling to bother physician	2 (0.7)
C4.7	Patient unwilling to adapt life-style	2 (0.7)
C4.8	Burden of therapy	1 (0.3)
C4.10	Patient takes food that interacts with drugs	1 (0.3)
C5	**Logistics**	**5 (1.7)**
C5.2	Prescribing error (only in case of slip of the pen)	5 (1.7)
C6	**Other**	**4 (1.4)**
C6.2	No obvious cause	4 (1.4)

Note: Only causes with the frequency of more than one were included.

### Factors Associated with DRPs in BPH Patients

Table 7 reveals the parameters that were significantly associated with the occurrence of DRPs in patients with BPH. Being elderly (p = 0.005), hospitalization for more than six days (p = 0.001), receiving polydrug therapy (p<0.001), and having multiple comorbidities (p<0.001) appeared to have significant associations with the occurrence of DRPs. Apart from that, several concurrent chronic diseases such as cardiovascular disease (p = 0.011), diabetes mellitus (p = 0.001), hypertension (p<0.001), and renal impairment (p = 0.011) were associated with the DRPs identified. On the other hand, factors such as BPH complications, use of α-blockers, use of 5-ARIs, and comorbidities such as dyslipidemia, erectile dysfunction, bronchial asthma, gouty arthritis and impairment had no significant association with the occurrence of DRPs.

**Table 7:** Factors associated with the occurrence of DRPs in BPH patients (n = 197).

Factors	DRPs (n = 197)		
	Yes	No	p-value
**Elderly**			
Yes	167 (84.8)	42 (67.7)	0.005^a^ *
No	30 (15.2)	20 (32.3)	
**Duration of hospitalization (more than six days)**			
Yes	79 (40.1)	10 (16.1)	0.001^a^ *
No	118 (59.9)	52 (83.9)	
**Polypharmacy**			
Yes	164 (83.2)	28 (45.2)	<0.001^a^ *
No	33 (16.8)	34 (54.8)	
**Multiple comorbidities**			
Yes	186 (94.4)	41 (66.1)	<0.001^a^ *
No	11 (5.6)	21 (33.9)	
**Cardiovascular disease**			
Yes	67 (34.0)	10 (16.1)	0.011^a^ *
No	130 (66.0)	52 (83.9)	
**Diabetes mellitus**			
Yes	100 (50.8)	16 (25.8)	0.001^a^ *
No	97 (49.2)	46 (74.2)	
**Hypertension**			
Yes	148 (75.1)	29 (46.8)	<0.001^a^ *
No	49 (24.9)	33 (53.2)	
**Renal impairment**			
Yes	43 (21.8)	4 (6.5)	0.011^a^ *
No	154 (78.2)	58 (93.5)	

a Computed using Continuity Correction.

* Statistically significant (p<0.05).

In addition to that, Table 8 and 9 tabulate the associations between different factors and the six domains of DRPs. It was found that there were significant associations between the occurrence of adverse drug reactions and parameters such as being elderly (p = 0.030), duration of hospitalization of more than six days (p<0.001) and polydrug therapy (p = 0.048). Also, six factors including being elderly (p<0.001), length of stay (p = 0.015), polydrug therapy (p<0.001), multiple comorbidities (p<0.001), diabetes mellitus (p = 0.039) and renal impairment (p = 0.001) were associated with drug choice problems.

**Table 8:** Factors associated with the occurrence of adverse reactions, drug choice problems and dosing problems.

Factors	Adverse reactions (n = 31)			Drug choice problems (n = 120)			Dosing problems (n = 50)		
	Yes	No	*p*-value	Yes	No	*p*-value	Yes	No	*p*-value
**Elderly**									
Yes	30 (96.8)	179 (78.5)	0.030^a^ *	109 (90.8)	100 (71.9)	<0.001^a^ *	46 (92.0)	163 (78.0)	0.040^a^ *
No	1 (3.2)	49 (21.5)		11 (9.2)	39 (28.1)		4 (8.0)	46 (22.0)	
**Duration of hospitalization**									
Yes	21 (67.7)	68 (29.8)	<0.001^a^ *	51 (42.5)	38 (27.3)	0.015^a^ *	23 (46.0)	66 (31.6)	0.078^a^
No	10 (32.3)	160 (70.2)		69 (57.5)	101 (72.7)		27 (54.0)	143 (68.4)	
**Polypharmacy**									
Yes	28 (90.3)	164 (71.9)	0.048^a^ *	104 (86.7)	88 (63.3)	<0.001^a^ *	46 (92.0)	146 (69.9)	0.002^a^ *
No	3 (9.7)	64 (28.1)		16 (13.3)	51 (36.7)		4 (8.0)	63 (30.1)	
**Multiple comorbidities**									
Yes	30 (96.8)	197 (86.4)	0.144^b^	115 (95.8)	112 (80.6)	<0.001^a^ *	48 (96.0)	179 (85.6)	0.079^a^
No	1 (3.2)	31 (13.6)		5 (4.2)	27 (19.4)		2 (4.0)	30 (14.4)	
**Cardiovascular disease**									
Yes	15 (48.4)	62 (27.2)	0.027^a^ *	41 (34.2)	36 (25.9)	0.188^a^	20 (40.0)	57 (27.3)	0.110^a^
No	16 (51.6)	166 (72.8)		79 (65.8)	103 (74.1)		30 (60.0)	152 (72.7)	
**Diabetes mellitus**									
Yes	18 (58.1)	98 (43.0)	0.164^a^	62 (51.7)	54 (38.8)	0.039^a^ *	31 (62.0)	85 (40.7)	0.010^a^ *
No	13 (41.9)	130 (57.0)		58 (48.3)	85 (61.2)		19 (38.0)	124 (59.3)	
**Hypertension**									
Yes	25 (80.6)	152 (66.7)	0.173^a^	87 (72.5)	90 (64.7)	0.229^a^	36 (72.0)	141 (67.5)	0.653^a^
No	6 (19.4)	76 (33.3)		33 (27.5)	49 (35.3)		14 (28.0)	68 (32.5)	
**Renal impairment**									
Yes	13 (41.9)	34 (14.9)	0.001^a^ *	33 (27.5)	14 (10.1)	0.001^a^ *	23 (46.0)	24 (11.5)	<0.001^a^ *
No	18 (58.1)	194 (85.1)		87 (72.5)	125 (89.9)		27 (54.0)	185 (88.5)	

a Computed using Continuity Correction;

b Computed using Fisher's Exact Test;

* Statistically significant (p<0.05).

**Table 9:** Factors associated with occurrence of drug use problems, drug interactions and other problems.

Factors	Drug use problems (n = 20)			Drug interactions (n = 97)			Other problems (n = 11)		
	Yes	No	*p*-value	Yes	No	*p*-value	Yes	No	*p*-value
**Elderly**									
Yes	16 (80.0)	193 (80.8)	1.000^b^	77 (79.4)	132 (81.5)	0.801^a^	10 (90.9)	199 (80.2)	0.696^b^
No	4 (20.0)	46 (19.2)		20 (20.6)	30 (18.5)		1 (9.1)	49 (19.8)	
**Duration of hospitalization**									
Yes	7 (35.0)	82 (34.3)	1.000^a^	40 (41.2)	49 (30.2)	0.095^a^	5 (45.5)	84 (33.9)	0.519^b^
No	13 (65.0)	157 (65.7)		57 (58.8)	113 (69.8)		6 (54.5)	164 (66.1)	
**Polypharmacy**									
Yes	15 (75.0)	177 (74.1)	1.000^a^	87 (89.7)	105 (64.8)	<0.001^a^ *	8 (72.7)	184 (74.2)	1.000^b^
No	5 (25.0)	62 (25.9)		10 (10.3)	57 (35.2)		3 (27.3)	64 (25.8)	
**Multiple comorbidities**									
Yes	17 (85.0)	210 (87.9)	0.722^b^	96 (99.0)	131 (80.9)	<0.001^a^ *	8 (72.7)	219 (88.3)	0.142^b^
No	3 (15.0)	29 (12.1)		1 (1.0)	31 (19.1)		3 (27.3)	29 (11.7)	
**Cardiovascular disease**									
Yes	8 (40.0)	69 (28.9)	0.429^a^	41 (42.3)	36 (22.2)	0.001^a^ *	3 (27.3)	74 (29.8)	1.000^b^
No	12 (60.0)	170 (71.1)		56 (57.7)	126 (77.8)		8 (72.7)	174 (70.2)	
**Diabetes mellitus**									
Yes	12 (60.0)	104 (43.5)	0.234^a^	52 (53.6)	64 (39.5)	0.038^a^ *	5 (45.5)	111 (44.8)	1.000^b^
No	8 (40.0)	135 (56.5)		45 (46.4)	98 (60.5)		6 (54.5)	137 (55.2)	
**Hypertension**									
Yes	14 (70.0)	163 (68.2)	1.000^a^	89 (91.8)	88 (54.3)	<0.001^a^ *	8 (72.7)	169 (68.1)	1.000^b^
No	6 (30.0)	76 (31.8)		8 (8.2)	74 (45.7)		3 (27.3)	79 (31.9)	
**Renal impairment**									
Yes	2 (10.0)	45 (18.8)	0.545^b^	21 (21.6)	26 (16.0)	0.334	3 (27.3)	44 (17.7)	0.425
No	18 (90.0)	194 (81.2)		76 (78.4)	136 (84.0)		8 (72.7)	204 (82.3)	

a Computed using Continuity Correction;

b Computed using Fisher's Exact Test;

* Statistically significant (p<0.05).

Besides that, patients who were elderly (p = 0.040), received polydrug therapy (p = 0.002), and had comorbid diseases such as diabetes mellitus (p = 0.010) and renal impairment (p<0.001) were more susceptible to the occurrence of dosing problems. In contrast, five factors were found to be significantly associated with drug interactions, including polydrug therapy (p<0.001), multiple comorbidities (p<0.001), cardiovascular disease (p = 0.001), diabetes mellitus (p = 0.038), and hypertension (p<0.001). Other factors had no statistically significant association with DRPs such as drug use problems and “other” problems.

## Discussion

### DRPs in BPH Patients

This is the first study to investigate drug-related problems among patients with BPH. Although PCNE classification has been tested for its validity, discrepancies in the classification of DRPs can occur, depending largely on factors such as the investigator's clinical experience with DRPs, study design, and study setting [30].

A total of 390 DRPs were identified, and there was an average of 1.5±1.3 DRPs per hospital admission. Drug choice problems, drug interactions and dosing problems were reported as the most frequently encountered DRPs in this study. On the contrary, a similar study conducted on geriatric patients in Taiwan reported an average of 2.2±1.6DRPs per patient and that the most commonly reported DRPs were drug use problems, drug choice problems, and drug interactions [6]. Discrepancies between these studies suggest that different health care settings and study populations may lead to differential classification of DRPs.

### Adverse Reactions

A review conducted by Hanlon *et al.* demonstrated that, of all medications prescribed to elderly patients, hypoglycemic agents, anticoagulants, cardiovascular agents, diuretics, and analgesics were the most common classes of medications that were associated with preventable adverse reactions [36]. Similarly, antidiabetic agents, including insulin and oral hypoglycemic agents were seen to cause hypoglycemia in this study. This was also consistent with a local study conducted on diabetic patients with comorbid hypertension [32].Apart from that, antiplatelet agents and anticoagulants were found in this study to cause bleeding disorders, whereas antihypertensive agents including ACE-inhibitors and diuretics were associated with persistent dry cough, hyponatremia, and gynaecomastia, respectively [37], [38]. In addition to that, α-blockers were found to be commonly correlated with vasodilatory adverse effects such as syncope and postural hypotension, and this was clearly manifested in this study [39]. Therefore, BPH patients, especially those who are elderly, should be frequently monitored to reduce the risk of adverse drug reactions [12].

### Drug Choice Problems

Drug choice problems were the most frequently identified DRPs in this study. More than one-third of the drug choice problems described in the Updated Beers Criteria were detected [34]. In our sample, 20.4% of the hospital admissions were associated with prescriptions of inappropriate medications, a number that was very close to that of a European study [40]. However, the number of cases associated with inappropriate prescriptions was much greater than a recent study conducted locally, which reported that only 8.8% of patients received inappropriate medications [32]. Apart from that, approximately 12% of all the cases of hospitalizations were found to be associated with insufficient medication. This prevalence was much less than that reported by Tulner *et al.*, in which nearly one third of the study patients were identified to have untreated indications [41]. Majority of the cases was correlated with unattended anemia especially in patients with renal impairment. This was consistent with a study conducted in the United States, which reported that 23% of the study population had unrecognized anemia [42]. Also, the number of cases correlated with the use of drugs without indication, involving overutilization of gastroprotective agents and antiemetics, and was found to be greater than that reported in a local study [32]. The high prevalence of drug choice problems in this study indicates the need for pharmacists to review the medications prescribed to elderly patients, to reduce the risk of inappropriate use of medications.

### Dosing Problems

Overdosing of medications in renal impaired patients was found to be the most frequently identified dosing problem. The prevalence of this dosing problem in our sample was four times greater than that from a study conducted in Singapore [43]. The authors of another study hypothesized that serum creatinine levels are less accurate in predicting actual renal function, and therefore, they lead to unrecognized renal impairment, especially in elderly patients. Also, most physicians do not calculate patients' creatinine clearance and therefore neglect the necessity to adjust medication dosages [44]. In this study, ranitidine was most commonly implicated in dosing problems, especially in patients with renal impairments. These results are similar to that of another study conducted at the same hospital setting [32]. Manlucu *et al.* recommended in their review that the dose of ranitidine should be reduced in patients with impaired renal functions [45]. This was probably because ranitidine was being excreted unchanged through the renal route and there was an increase in the serum concentration and half-life of ranitidine with decreasing renal functions [45].

Therefore, it is of the utmost importance that pharmacists should be involved in medication reviews, to ensure that the appropriate dose of drugs is delivered to patients, particularly those with decreased renal functions.

### Drug Use Problems

In this study, 6% of cases were associated with drug use problems, where patients were not compliant to their medications. Of these, BPH medication itself was implicated in the majority of the drug use problems. However, the compliance status for BPH treatment in this study was much greater than that reported in other studies. Nichol *et al.* reported that only 40% of patients adhered to their BPH medications [46]. However, the Triumph Project conducted in Europe reported better patient adherence to BPH medications, in which compliance to α-blockers and 5-ARIs was 67% and 73%, respectively [47].Poor medication compliance in BPH patients may be because BPH is not a life-threatening disease, and patients may be especially likely to not comply with their medications if they have not experienced LUTS [46]. In addition, α-blockers are associated with undesirable side effects such as dizziness and hypotension, which may cause patients to discontinue treatment [39], [46].

Unrecorded information regarding patients' compliance in the medical records in this study may have led to discrepancies in the reported prevalences between studies. This could lead to underestimation of DRPs.

### Drug Interactions

A quarter of the DRPs we identified were made up of potential drug-drug interactions. The number of drug interactions detected was only half of that from a study of geriatric patients [43]. In the present study, highly prescribed medications including α-blockers, amlodipine, simvastatin, aspirin and clopidogrel were implicated in most cases of drug interactions. The finding was different from research conducted by Koh *et al.* in which beta-blockers, non-steroidal anti-inflammatory agents (NSAIDs), and ACE inhibitors were most commonly involved in drug-drug interactions [43]. The interactions between α-blockers and CCBs were the most prevalent drug interactions in this study. This was attributed to the mechanisms of action of both classes of drugs, which will lead to excessive reductions in blood pressure [48].

The judgment of drug-drug interactions was on the basis on an established literature and standard references. The combinations of offending drugs will still be used in certain hospital settings, depending on their prescription policies. Therefore, frequent monitoring should be conducted to minimize adverse effects secondary to drug-drug interactions.

### Causes of DRPs in BPH Patients

The most prevalent cause of DRPs in this study appeared to be “drug selection or dose selection”. This included the causes for DRPs such as adverse reactions, drug choice problems, dosing problems, and drug interactions. In contrast, drug use process was matched with DRPs such as “drug not taken/administered”, in which patients did adhere to their medications. However, the number of DRPs varied from the number of causes of DRPs, as there were conditions where several DRPs were matched with a single cause. This is consistent with a study conducted on geriatric patients in Taiwan [6]. The assignment of causes to each DRP was based on researcher's own judgment or from information obtained from the medical records, which could lead to difficulties in assessing the causes of DRPs, as some possible causes of DRPs may not be retrievable from medical records.

### Factors Associated with DRPs in Elderly BPH Patients

Consistent with other studies, being elderly was shown to be associated with an increased prevalence of DRPs [6], [36]. Of the six problem domains examined, advanced age was significantly associated with the occurrence of adverse drug reactions, drug choice problems, and dosing problems. The high prevalence of DRPs among elderly patients may be explained by the fact that elderly people are more vulnerable to have multiple comorbidities, because of age-related deterioration of health and organ functions [49]. Also, geriatric patients were found to be disproportionately likely to receive polydrug therapy and inappropriate prescriptions, which can subsequently lead to drug choice problems and dosing problems [34]. This was supported by a local study conducted in Hospital Universiti Sains Malaysia (HUSM), which reported that the prevalence of medication errors was high among geriatric patients [50]. Nevertheless, this finding was contradicted by other data, which indicated that being elderly did not result in a higher risk of experiencing DRPs [32], [43].

### Duration of Hospitalization

In the present study, we found that patients who stayed for more than six days in the ward were at greater risk of experiencing adverse reactions and drug choice problems. However, a local study reported conflicting findings, in which hospitalization for less than a week was significantly associated with drug choice problems [32]. Also, a significant correlation between the duration of hospitalization and the other problem domains was not detected in this study. These findings are contrary to other global studies [51], [52]. Firstly, a study by Moura *et al.* revealed comparable findings, in which patients who were hospitalized for a longer period were more susceptible to drug-drug interactions [51]. Also, a study conducted on hospitalized cancer patients revealed a significant association between length of hospitalization and drug interactions [52].

That these findings are contradictory may be because of confounding factors such as the severity of illness and the number of medications prescribed to the patients [51]. Besides that, the small sample size recruited in this study may have reduced its generalizability. Further studies should be carried out to investigate the association between the duration of hospitalization and occurrence of DRPs.

### Polydrug therapy

Polydrug therapy appears to be a common condition among elderly patients, because of the presence of multiple comorbidities that require chronic medical therapies [43], [53]. It was not surprising that polydrug therapy was found to be significantly associated with the occurrence of DRPs in this study. From the literature reviewed, it had been strongly supported that an increase in medications prescribed can result in an increased risk of medication errors, which can subsequently lead to DRPs [26], [49], [50], [54]. At a more detailed level, patients who were prescribed more than five medications tended to experience adverse reactions, drug choice problems, dosing problems, and drug interactions. The findings in this study were consistent with that from a study conducted by Hajjar *et al.*, which showed that the use of multiple chronic medications can lead to inappropriate prescriptions and adverse drug reactions [26]. Also, Viktil and colleagues demonstrated that receiving polydrug therapy is correlated with a heightened risk of drug interactions [55].

Therefore, healthcare providers and especially pharmacists should be aware of the consequences of providing polydrug therapy to patients. More efforts should be put into medication review to minimize polydrug therapy whenever possible, to reduce the risk of DRPs.

### Multiple Comorbidities

Consistent with the present study, patients who had other comorbid diseases were found to a have higher risk of DRPs [26]. Because of the presence of multiple concurrent chronic conditions, patients with BPH are more likely to be prescribed multiple medications, leading to increased risk of DRPs [26]. Also, having multiple comorbidities has been shown to account for 26.5% of ADRs in the elderly [54]. However, this is not comparable with the findings of this study, which revealed that having multiple comorbidities was only significantly associated with drug choice problems and drug interactions, whereas correlation with other problem domains was not detected. These discrepancies are probably because of differences in research methods and tools used to assess DRPs.

Since it is inevitable that the majority of elderly patients present with multiple comorbid diseases, it is important to have medication reconciliation in all the health care settings to avoid medication errors including inappropriate prescriptions, dosing errors, and drug-drug interactions.

### Cardiovascular Disease

In this study, BPH patients with comorbid cardiovascular diseases were found to experience more DRPs. This was probably because patients with cardiovascular diseases often require multiple medications, which can subsequently lead to complications. Numerous studies have concluded that cardiovascular medications are the most common drug class associated with the occurrence of DRPs [6], [32], [56]. Also, the largest prospective study on adverse drug reactions in the United Kingdom reported that medications used to treat cardiovascular diseases such as anticoagulants, fibrinolytics, heparin, and diuretics were frequently implicated in causing adverse reactions [57]. Similarly, in the present study, we found that cardiovascular medications, particularly antiplatelet agents and lipid lowering agents, were associated with most cases of adverse reactions and drug-drug interactions.

The relationship between cardiovascular diseases and occurrence of DRPs has been established. The prevalence of DRPs among patients with cardiovascular diseases strongly supports the role of pharmacists in assuring patient safety.

### Diabetes Mellitus

Patients in our sample with diabetes mellitus were at a higher risk of having DRPs. This may be supported by a study showing that, on average, there were 4.6±1.7 DRPs per diabetic patient [29]. In addition to that, a local study on diabetic patients with comorbid hypertension revealed that more than 90% of the study population had at least one DRP [32].By comparing this across the problem domains, BPH patients with comorbid diabetes mellitus were at higher risk of acquiring drug choice problems, dosing problems, and drug interactions. In this study, antidiabetic agents such as sulphonylurea and metformin were involved in approximately 10% all the drug choice problems. This included the use of oral antidiabetic agents in patients with severe hepatic and renal impairment, which are contraindications [58]. Nevertheless, the data was much less robust than that from the other studies conducted [32]. This was probably because of the smaller sample size of diabetic patients in this study.

### Hypertension

Surprisingly, BPH patients with comorbid hypertension were significantly more likely to experience drug interactions in the present study. This phenomenon could be explained by the wide use of antihypertensive agents, particularly calcium channel blockers, which is a commonly used drug category that can have drug-drug interactions with α-blockers and statins [48]. Likewise, a local study reported that 16% of the diabetic patients with comorbid hypertension were on drugs with the potential for drug-drug interactions [32]. Thus, the association between being hypertensive and occurrence of DRPs is concerning and pharmacist's are vital in recognizing potential drug-drug interactions and reducing the risk of DRPs.

### Renal Impairment

Renal impairment was found to be significantly associated with the occurrence of DRPs. Consistently, a local study on diabetic patients reported that patients with impaired renal functions were more susceptible to drug choice problems and dosing problems [32]. Besides that, Hajjar *et al.* showed that renal insufficiency contributed to 5.2% of adverse reactions among elderly patients [54]. Stemer and Lemmens-Gruber further elucidated this by showing that patients with decreased renal functions are often suffering from complications such as electrolyte imbalances, anemia, and other metabolic syndromes, and these complications were found to be untreated in the present study [59]. Apart from that, renally impaired patients, especially those who are on renal replacement therapy, are prone to DRPs because of changes in the pharmacokinetics of drugs [59]. In this study, the risk of getting DRPs was further heightened by factors such as being elderly, having multiple comorbidities, and receiving polydrug therapy. Also, it was found that renal impairments among elderly patients are often left unrecognized, and therefore dosage adjustments of drugs are not undertaken [44].

Based on these findings, health care providers should prevent DRPs among patients with impaired renal functions, to reduce the risk of drug-related morbidity and mortality.

### Study Limitations

This study has several limitations and shortcomings. The first limitation concerns the retrospective nature of this study using medical records, established references, and a literature review. The prevalence of DRPs might be underestimated, as some important data including physicians' and patients' perceptions could not be found in the medical records. Furthermore, it was difficult to obtain some relevant clinical data such as patients' body weight, the duration since BPH was diagnosed, and patients' compliance to medications. Also, selection bias might be a factor because of the presence of missing data.

The second limitation is that the Hospital Information System (HIS) was only able to generate the list of BPH patients who were registered from 1^st^ January 2009 to 30^th^ June 2012. Therefore, the findings of this study may not generalize to the actual population of Malaysia, because of the relative small sample size. Finally, this study used only part of the PCNE Classification in assessing DRPs. Interventions by physicians as well as the outcome of interventions listed in the tool were not included.

## Conclusion

Of all the BPH medications, α-blockers were the most frequently prescribed medication among the study population. Monotherapy was commonly used as the first line treatment, compared to dual therapy and triple therapy. Nonetheless, concurrent medications that were most frequently implicated in this study were antihypertensive agents, antibiotics, and statins.

In conclusion, the prevalence of DRPs is high among patients with BPH. The most commonly identified problems were drug choice problems, drug interactions, and dosing problems. On the other hand, several factors were found to be associated with the occurrence of DRPs, including being elderly, duration of hospitalization, receiving polydrug therapy, and having multiple comorbidities. Also, comorbid diseases such as cardiovascular disease, diabetes mellitus, hypertension, and renal impairments were reported to be significantly predictive of the occurrence of DRPs. The identification of subtypes of DRPs among BPH patients and the factors associated with DRP may facilitate risk reduction strategies.
